# Dutch DALYs, current and future burden of disease in the Netherlands

**DOI:** 10.1186/s13690-020-00461-8

**Published:** 2020-09-22

**Authors:** Henk B. M. Hilderink, Marjanne H. D. Plasmans, M. J. J. C. (René) Poos, Petra E. D. Eysink, Ronald Gijsen

**Affiliations:** National Institute for Public Health and the Environment (RIVM), P.O. Box 1, 3720 BA Bilthoven, the Netherlands

**Keywords:** Dutch DALYs, National Burden of disease, Future projections

## Abstract

**Background:**

The Disability Adjusted Life Year (DALY) is a measure to prioritize in the public health field. In the Netherlands, the DALY estimates are calculated since 1997 and are included in the Public Health Status and Foresight studies which is an input for public health priority setting and policy making. Over these 20 years, methodological advancements have been made, including accounting for multimorbidity and performing projections for DALYs into the future. Most important methodological choices and improvements are described and results are presented.

**Methods:**

The DALY is composed of the two components years of life lost (YLL) due to premature mortality and years lost due to disability (YLD). Both the YLL and the YLD are distinguished by sex, age and health condition, allowing aggregation to the ICD-10 chapters. The YLD is corrected for multimorbidity, assuming independent occurrence of health conditions and a multiplicative method for the calculation of combined disability weights. Future DALYs are calculated based on projections for causes of death, and prevalence and incidence.

**Results:**

The results for 2015 show that cancer is the ICD-10 chapter with the highest disease burden, followed by cardiovascular diseases and mental disorders. For the individual health conditions, coronary heart disease had the highest disease burden in 2015. In 2040, we see a strong increase in disease burden of dementia and arthrosis. For dementia this is due to a threefold increase in dementia as a cause of death, while for arthrosis this is mainly due to the increase in prevalence.

**Conclusions:**

To calculate the DALY requires a substantial amount of data, methodological choices, interpretation and presentation of results, and the personnel capacity to carry out all these tasks. However, doing a National Burden of Disease study, and especially doing that for more than 20 years, proved to have an enormous additional value in population health information and thus supports better public health policies.

## Background

The disability-adjusted life years (DALY) is a measure to prioritize in the public health field. The DALY combines health indicators - mortality, morbidity and the severity of health conditions - in one measure and represents the health loss or gap to perfect health in a population. Since the concept of the Burden of Disease (BoD) was introduced in the 1990s [1], the National Institute for Public Health and the Environment in the Netherlands (RIVM) adopted and applied the DALY in national burden of disease studies (NBoD), the first one being published in the 1997 Public Health Status and Foresight report [2, 3]. Ever since, NBoD estimates have been included in all following editions which became a periodic reporting to the Dutch ministry of Health, Welfare and Sports [4–8]. These studies serve as an input for public health priority setting and policy making.

During the more than 20 years of NBoD history, the methodology has evolved. For example, the notion that multimorbidity is an issue of growing concern in public health, especially in an ageing population, asks for a correction of YLD, in order to properly support policy intervention strategies. Also, the improvement of data availability and quality allows to include more health conditions in recent NBoDs. In addition, the results are presented more clearly and effectively with graphical presentations, instead of long tables in paper reports. Moreover, the more recent NBoD estimates are not only done for the present situation, or the most recent years for which data are available, but also for the future. These changes resulted in a web publication with estimates for the period 2015–2040, based on data for a total 101 health conditions and corrected for multimorbidity [9].

The objective of this article is to give an overview of the methodological choices and improvements made for the Dutch NBoD study and to present the results for the Public Health Foresight Study 2018 in more detail. This hopefully helps and inspires more countries to do their own NBoDs.

### Methodology

The burden of disease quantifies the health loss in a population. It is expressed in DALYs, a so-called summary measure of population health. This measure combines the premature mortality due to a specific cause of death, expressed in Years of Life Lost (YLL), and the occurrence of health conditions in a population, weighted for the severity of the health conditions, expressed in Years Lost due to Disability (YLD). The YLL are calculated by summing the number of deaths, multiplied by the number of years of life remaining at the age at which death occurs. The YLD combine a weighting factor or disability weight for a health condition with the number of incident or prevalent cases. For each health condition, the YLL and the YLD are distinguished by sex and 21 age groups (i.e. 0 to 1 year, 1 to 5 years, the 5 to 95 years distinguished in 5 year age groups and the 95+ age group). The sum of the YLL and the YLD results in the DALY. Though the methodology to calculate DALYs might look relatively simple [10], there is no common, unambiguous method to operationalize the underlying concepts YLL and YLD.

### Methodological choices

To be able to calculate the Burden of Disease in a population, various choices have to be made, for example regarding the selection of health conditions, a prevalence or incidence approach and which life tables to use. Next, these calculations require a rather large data demand, and often different types of data sources are used (e.g. registrations and surveys) which may imply potential inconsistencies. Most important methodological choices in doing the Dutch NBoD study are elaborated on, and overview of relevant data sources is given.

### Selection of health conditions

The first Dutch NBoD estimates started with a selection of 52 health conditions which were extensively described [11]. Selection criteria were based on the contribution to the total morbidity and/or (premature) mortality in the population, the extent to which conditions are avoidable and on related health care costs [12]. In subsequent studies, additional conditions were added, based on the impact they have on the individual and on the society, the number of hospitalisations, and the obstacles to social participation encountered by people suffering from chronic diseases or disorders [13]. This selection included among others infectious diseases, acute and chronic diseases, congenital anomalies and injuries. The most recent selection was expanded to 74 specific conditions, and 17 so-called residual disease categories representing the remainder of health conditions within the 17 ICD-10 chapters [14], resulting in 91 disease categories [15]. This expansion ensured compatibility with and possibility of aggregation to the commonly-used ICD-10 chapters. This aggregation leaves less room for arbitrariness which is the case with a selection at the more detailed level.

The progression, severity and consequences of individual health conditions are often of heterogeneous nature. By dividing diseases into disease stages or sequelae, more homogeneity can be created. Disease stages can be defined on the basis of steps in the disease progression in time or as grades/levels of disease severity. Within these disease stages, suffering of patients and consequences for the functioning in domains of health, like mobility, cognition, mentally and autonomy, treatment options and prognosis are more or less comparable. For this reason, it is often desirable to provide more than one weighting factor per disease.

### Registration or population level

One of the choices is regarding the study population to be considered. Are all cases considered, or only cases utilizing medical care and therefore registered in the health care system? Preferably, a burden of disease study intends to cover the whole burden of both diagnosed and undiagnosed health conditions. In studies on infectious and parasitic diseases, population incidence rates are often calculated, based on surveillance data [16]. However, in studies which include different types of conditions, the calculation of the burden of disease is often based on health care registrations. Health care registration DALYs might not always reflect population DALYs because these do not include the morbidity of undiagnosed conditions. For the Dutch DALYs, the burden for several health conditions is based upon health care registrations which might represent to a certain extent the population level, for example regarding the cancer registration. For particular health conditions such as depression and anxiety disorders, the estimates are at the population level, since these are based on epidemiological population studies. For other health conditions such studies are lacking, and estimates are based on health care registrations. This results in a hybrid approach of registration and population DALYs.

### Prevalence versus incidence

A major choice for calculating DALYs is whether an incidence or prevalence approach is used [17]. For the YLL, there seems to be wide consensus for applying an incidence approach that calculates the years lost over a person’s expected life (e.g. represented by the average remaining life expectancy). This incidence approach to YLL has been applied in all Dutch NBoD studies. For the YLD, different approaches are applied, namely an incidence approach where incidence of health condition and its duration are combined, versus a prevalence approach, taking the (point) prevalence of a disease at a certain moment. Although incidence and prevalence are related to each other since the current prevalence is the result of the past incidence, recovery and mortality, there are distinct differences between the two approaches. These concern 1) different objectives of the study at hand and 2) different nature of health conditions. If the objective is to provide a “picture” of the health situation at a certain moment in time, a prevalence approach might be most useful. This approach is often applied to chronic and long-lasting health conditions. The incidence approach is more related to the future burden of disease resulting from incident cases and is often applied to infectious and parasitic diseases (e.g. [18]) and injuries. The incidence approach can therefore be used to analyse intervention strategies [17]. The Dutch NBoDs always applied a prevalence approach.

### Disability weights

A weighting factor or disability weight for a health condition is a measure of the severity of the consequences of a health condition for the physical, psychological and social functioning of patients, on a scale from 0 (no effects) to 1 (very serious adverse effects corresponding with death). For the Netherlands, specific disability weights were derived in the Dutch Disability Weight Study [19]. In this study, medical experts valuated disease stages on the basis of combinations of diagnoses and generic health descriptions according to the EQ-5D instrument for generic health [20, 21]. Within the EQ-5D system, every dimension has three levels of functioning, i.e. no problems, some problems or extreme problems. In the Dutch Disability Weight Study, the original five dimensions of EQ-5D, mobility, self-care, usual activities, pain / discomfort, anxiety / depression were expanded with a sixth dimension, namely cognition. This resulted in disability weights for every stage of specific health conditions. For each health condition, we obtained an average disability weight by combining the disability weights from the Dutch Disability Weight Study with the distribution of the stages also referred to as severity distribution [22]. The disability weights and severity distributions being used in all Dutch NBoD studies originate from these studies.

### 100% morbidity

Where all deaths are allocated to a certain cause of death, and therefore YLL represents 100% of the mortality, this is not the case for morbidity. A selection of the 74 health conditions implies a less than 100% representation of the total morbidity, since obviously prevalence data are not available for the 17 residual disease categories in every chapter of the ICD-10. It is relevant to have estimates of the total burden of disease, and not only for a selection of diseases, since it is being used as a denominator, for example to express the share of a disease or of a risk factor as a percentage of the total burden. The applied methodology to calculate the YLD for 17 residual disease categories, follows Mathers et al. [23]. In this approach, we first aggregate the YLL and YLD to the ICD-10 chapter level for the health conditions included in the selection. Next, we calculate the specific ratios of these aggregated YLL and YLD for each ICD-10 chapter. In the last step, we multiply these ratios with the YLL of the 17 residual disease categories. This approach implicitly assumes that the ratio of the YLL and YLD for the health conditions in each ICD-10 chapter are representative for those not included within the ICD-10 chapter.

### Multimorbidity

Taking multimorbidity (i.e. the co-occurrence of multiple chronic or acute diseases and health conditions within one person [24]) into account, is becoming more relevant, especially when addressing future trends [8]. However, various BoD estimates do not correct for multimorbidity which can lead to inaccuracies in BoD estimations, particularly in ageing populations that include large proportions of persons with two or more health conditions [25]. If multimorbidity is not accounted for and health conditions are considered separately, the so-called additive method is implicitly assumed, in which disability weights are summed. The combination of different health conditions can result in disability weights exceeding 1. Correcting for multimorbidity can lead to a (downward) adjustment of the YLD up to 26% when the maximum limit method is used for the combined disability weights [25]. Until 2018, Dutch estimates did not include corrections for multimorbidity. In 2018, a multiplicative method for combined disability weights and assuming independent occurrence of combinations of up to five different health conditions was applied [8]. This was done for every 5-year age and sex group separately. This correction for multimorbidity impacts the DALYs of especially those health conditions with a relative high share of the YLD in the DALY, for example mental disorders and musculoskeletal diseases.

### Dutch life table

To calculate the YLL, one needs to have an estimate of the years of life that are lost at a certain age of death. A life table is normally used for this. A life table calculates the remaining life expectancy at specific ages, under the assumption that current age-specific mortality rates will be experienced throughout the rest of his or her life (the so-called period life expectancy). The choice which life table to use, depends on the purpose of the study. If you want to compare countries, a reference life table is a proper option. IHME uses the lowest world-wide observed age-specific mortality rates for their refence life table, representing an aspirational life expectancy level that all countries might be able to achieve [26]. Since the Dutch DALY is used as input for Dutch public health policy, current Dutch life tables have been chosen. Those might best represent the health loss in the Netherlands, and therefore, what potentially can be gained with policies.

### No redistribution of ill-defined cause of deaths

Several BoD studies have chosen to redistribute the so-called ill-defined cause of deaths, i.e. causes of death that are not specified as such or are considered to be wrongly allocated [27]. This concerns for example, specific cardiovascular diseases such as heart failure and cardiac arrest [28]. In the Dutch NBoD study no redistribution is applied. A redistribution might suggest a more precise depicting of the causes of death than we actually know. Moreover, it may reflect another source of uncertainty that we have to deal with when doing BoD estimates. If one chooses for redistribution of causes of death, also the morbidity data for the same conditions has to be redistributed (e.g. heart failure). If such a redistribution would be applied, such as done by IHME in the Global Burden of Disease (GBD) study [29], it would change causes of death resulting in a different ranking. For example, the number of deaths due to coronary heart disease will double after the IHME’s redistribution, and move to the first position as most important cause of death, while it is in the fourth according to the official reporting of Statistics Netherlands [30].

### Data

Mortality and cause of death data are obtained from the cause of death registry [31]. Several steps are taken before a cause of death is established. Firstly, the physician who determines the death has to describe the chain that leads to the death of a person on the death certification. Next, the automated coding software selects the underlying cause of death. This is done by Statistics Netherlands, using the ICD-10 rules for coding of the WHO [30]. For a part of the cases, the underlying cause of death cannot be selected automatically, so medical coders have to analyse the death certificates to assign the underlying cause of death [31]. The steps between registration by the physician and inclusion of an ICD-code in the Cause of Death registration are subject to errors or variation between comparable cases, especially the first step.

Preferably, data on prevalence and incidence come from epidemiologic population studies. However, these are scarce. Consequently, it is necessary to use data from health care registries. The NBoD used data of several types of registries, like the Netherlands Cancer Registry [32], the Nivel Primary Care Database [33], the Injury Surveillance System [34] and the National Hospital Care Basic Registration [35]. Especially the Nivel Primary Care Database is an important data source, because in the Netherlands General Practitioners have a good overview on the morbidity of the population [33]. The GP is the gatekeeper of specialist care which means that before a consultation in secondary care is possible, a referral by the GP is needed (except for emergency care). Besides, medical specialists report to GPs about their findings (e.g. diagnoses and treatments). However, not all persons with symptoms of a health condition have contact with the GP, so a part of the morbidity is not observed [36].

### Future projections

In the Public Health Foresight Study 2018, future projections were made. The projections in this study are based on observed trends for prevalence and incidence figures, and for causes of deaths. For prevalence and incidence, demographic projections (i.e. projections that are based on future changes to the size and age structure of the population while the relative sex-specific and age-specific rates from the base year of the projection are kept constant) are applied and where possible and relevant supplemented with epidemiological changes [15]. Causes of death are projected applying Poisson regression analyses on age- and sex-specific morality rates over the period 1996–2015, resulting in cause of death projections [37] which are consistent with the overall mortality prognoses [38]. The life tables of these prognoses are also used to calculate YLL in the future. For each year the applicable life table of that year is used.

## Results

The results of a BoD study are quite extensive, since the YLD, YLL and the DALY are distinguished by sex, 21 age categories, 91 health conditions and 17 ICD-10 chapters. On top of this, by doing projections, results are available for each year of the 2015–2040 period. To facilitate the use of the NBoD results for example by policy makers, notable attention is given to the presentation of the graphical results, in addition to providing results in a tabular form.

### Results for 2015

**Fig. 1:**
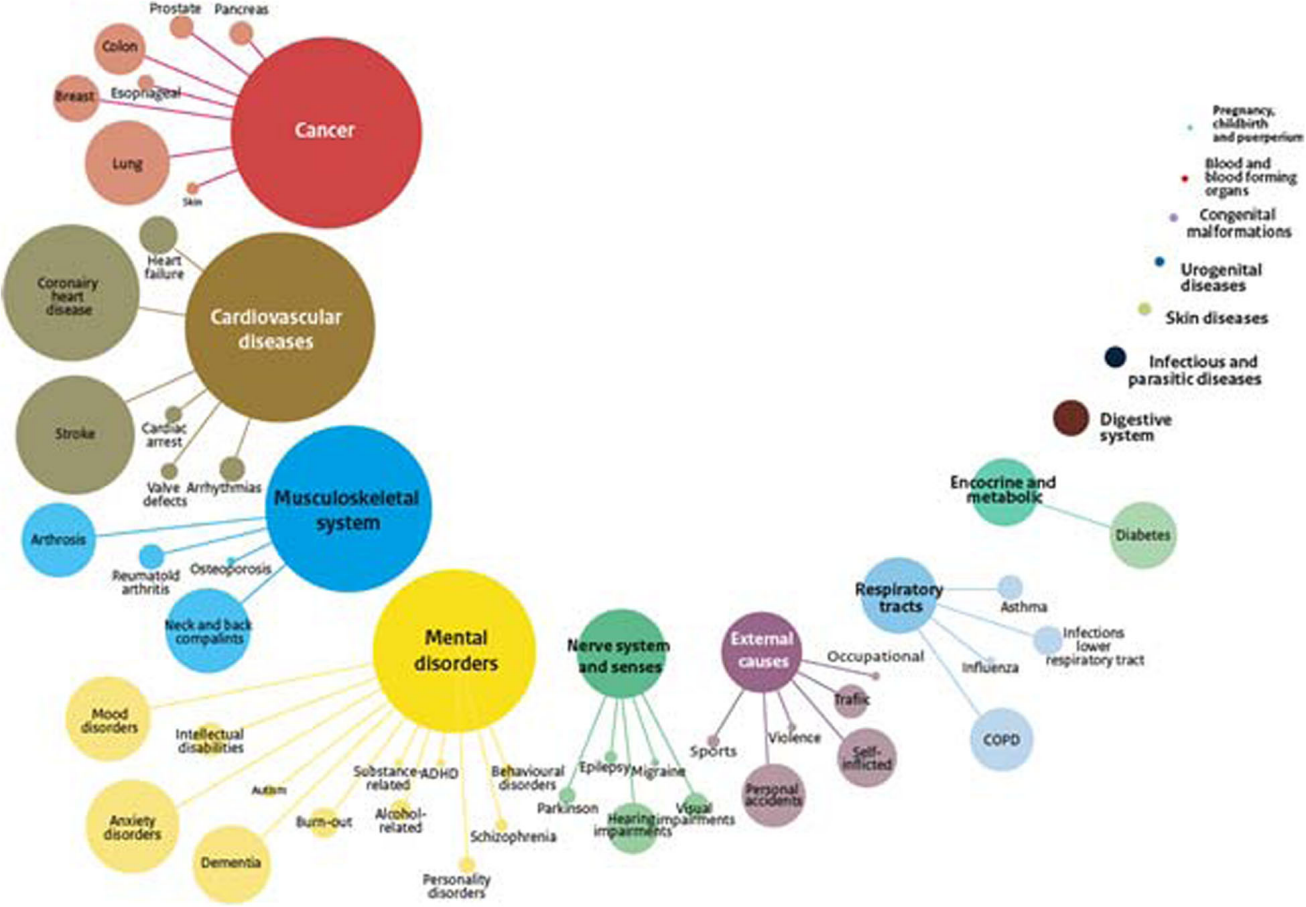
Burden of Disease in 2015, for 17 ICD-10 chapters and specific health conditions. Legend: The size of the bubbles represents the burden of disease, expressed in DALYs

In 2015, the total burden of disease in the Netherlands was almost 5 million DALYs. Of the seventeen ICD-10 chapters, cancer is the main cause of disease burden with 16.5% of the total Dutch burden of disease (Fig. 1). It is directly followed by the ICD-10 chapters cardiovascular diseases (16.3%), diseases of the musculoskeletal system (14.4%) and mental and behavioural disorders (14.0%). At the lower, more detailed level of health conditions, coronary heart disease had the highest disease burden with 5.3%, followed by stroke (4.6%), diabetes (3.8%), COPD (3.7%), anxiety disorders (3.5%) and lung cancer (3.4%).

**Fig. 2:**
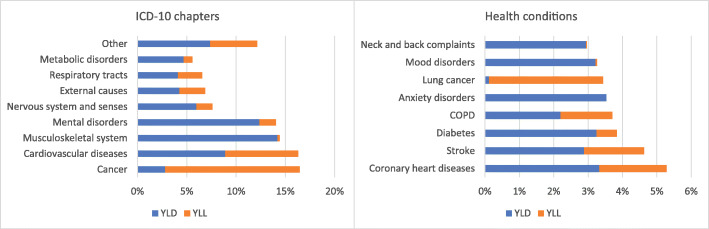
**a** and **b** Contribution of YLL and YLD to total burden of disease for ICD-10 chapters and specific health conditions, 2015. Abbreviations: COPD chronic obstructive pulmonary disease. Legend: Numbers are rounded to the nearest hundred

Looking in more detail at the two components provides insights in whether premature mortality (YLL) or disability (YLD) is most important in the disease burden. Overall, 65% of the disease burden is due disability, while 35% is due to premature mortality. At the ICD-10 chapter level, for cancer (malignant neoplasms) almost 5/6th (i.e. 14% of 17% in total) is due to YLL, while for musculoskeletal diseases and mental disorders, the picture is completely different and the YLD is the dominant component (Fig. 2). This is even more pronounced for specific health conditions. For example, for anxiety and mood disorders the contribution of YLD is almost 100%. In the case of lung cancer, almost the entire burden of disease is due to premature death; only 3.5% of the total number of DALYs is caused by a loss of healthy years.

### Projections in to the future

**Fig. 3:**
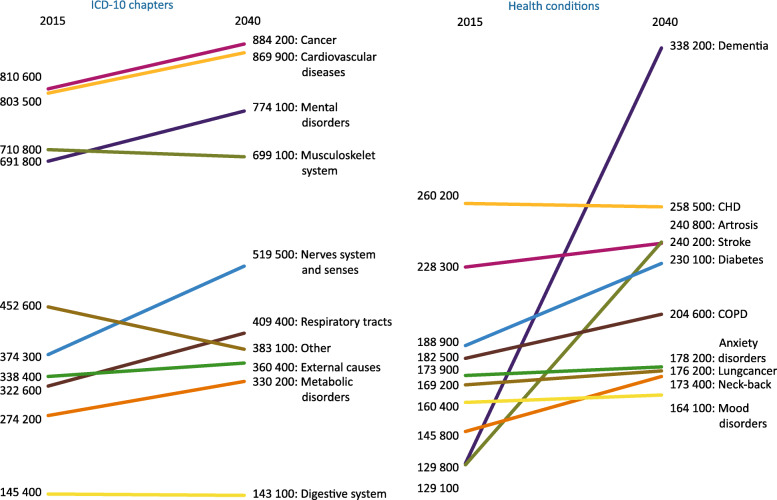
**a** and **b** DALY for ICD-10 chapters and specific conditions, 2015–2040. Abbreviations: CHD coronary heart diseases, COPD chronic obstructive pulmonary disease

Projections for the period 2015–2040 show that cancer and cardiovascular diseases remain the two ICD-10 chapters causing most disease burden (Fig. 3). In 2015, the third place was taken by diseases of the musculoskeletal system, while by 2040, the ICD-10 chapter mental disorders will have taken over the third place. The great increase during the period 2015–2040 in mental disorders and diseases of the nervous system and senses is mainly attributable to a large rise in deaths due to dementia. The increase in mental disorders is mainly due to a threefold increase in dementia as cause of death and a doubling of its prevalence over the period 2015–2040. The health conditions forming the top ten of disease burden do not change over the projection period. However, there are remarkable changes in the position of these top ten health conditions. In 2015, dementia just made it to this top ten while it has unmistakably taken over the first position in 2040. In 2040, coronary heart diseases and arthrosis will be in second and third place. Next to the increase of dementia in burden of disease, the increases of arthrosis and diabetes are remarkable, which can, at least, partly be linked to increases in overweight.

### Future DALYs by age

**Fig. 4:**
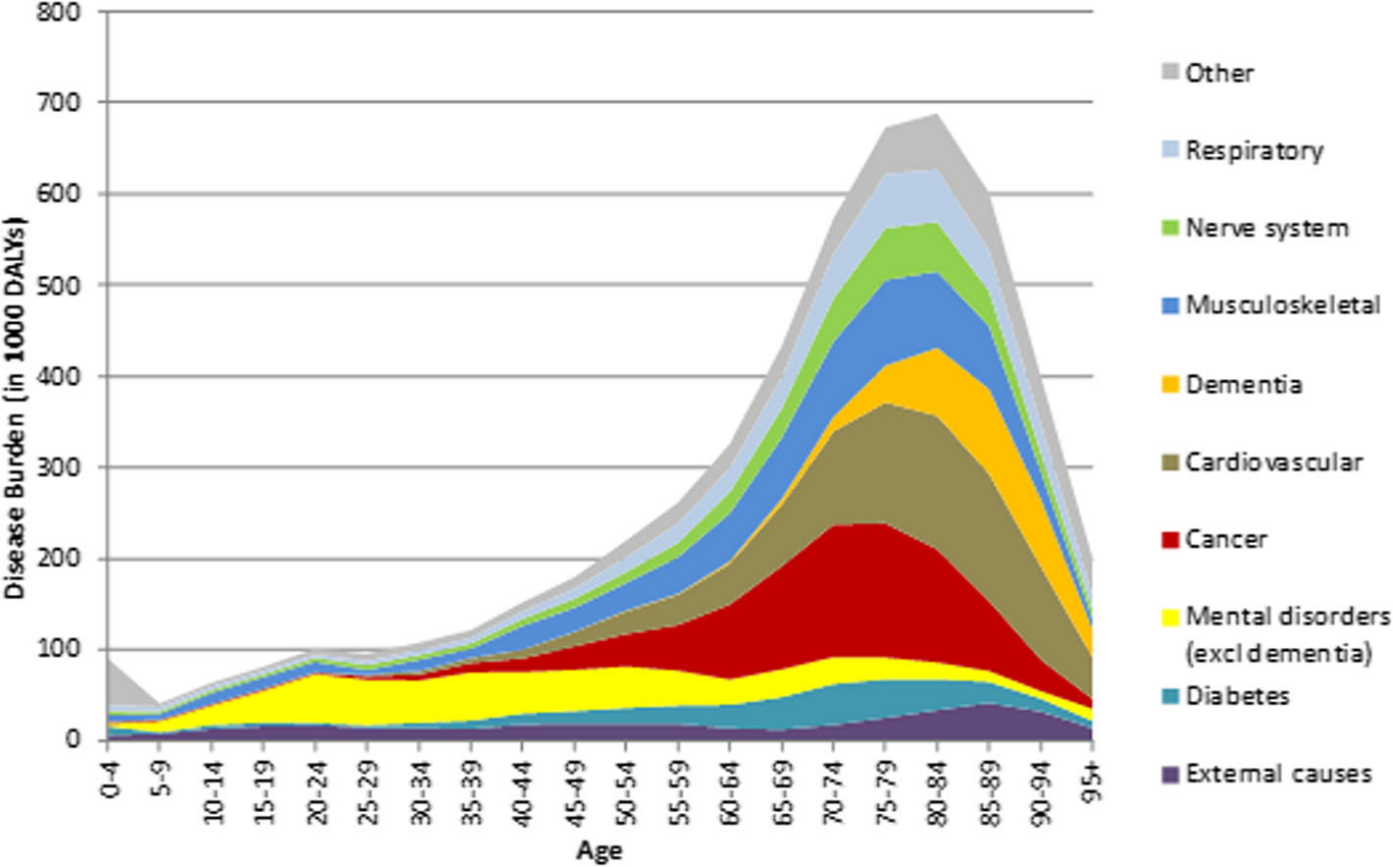
Dutch DALYs by age for selected health conditions, 2040

Since the DALY is a volume measure, i.e. having more people at older ages resulting in higher outcomes of old-age related health conditions such as dementia, it is also of additional value to look at the outcomes by age. In 2040, the peak of DALYs is, as expected, around 80 years, with cancer as important contributor just before that age, cardiovascular disease around that age, and dementia setting in after that age (Fig. 4). It also shows that at younger ages, i.e. before the age of 30, mental disorders and external causes of morbidity and mortality make up half of the disease burden.

## Discussion

One of the striking outcomes of Public Health Status and Foresight Study 2018 is a dramatic increase from 2015 to 2040 in burden of disease caused by dementia, a finding which was at the core of one of the missions of the Dutch Cabinet [39]. Another noteworthy observation is that the disease burden of cardiovascular diseases remains high over the period 2015–2040, though there is a shift in contribution to the disease burden from the YLL to the YLD. This reflects cardiovascular diseases becoming less lethal due to improvements in survival, but still cause a high burden through the YLD since heart conditions remain for the rest of your life.

The results of the recent Dutch NBoD are presented at different aggregation levels (i.e. specific health conditions, ICD-10 chapters and the total burden of disease). This leaves less room for interpretation of the results which was the case in previous Dutch BoD studies which had only a selection of specific health conditions. For example, when the ranking of health conditions depends on the level of detail in the selection of them. This is the case, among others, with dementia that can be caused by over fifty different brain disorders, which also do not all fall in the same ICD-10 chapter. Also being able to explore results for different ages allows to better understand possible future developments especially given the ageing of the population. The methodological choices that have been made and the data sources used for the Dutch NBoD differ from the choices made in other BoD studies, such as the GBD and other NBoD studies. Therefore, a comparison of the results with other studies is of limited value. More flexibility in the methodological choices, for example regarding life tables, disability weights or redistribution of ill-defined codes, would allow a better comparison with other BoD studies and should be strived for.

To be able to perform a National Burden of Disease study requires the availability of a wide range of data, both regarding morbidity and mortality. Even with these data available, calculating a DALY is not a straightforward exercise and involves various choices about data processing, methodological choices and presentation and interpretation of results. And even then, underlying uncertainties, not only statistical ones but also structural ones might be of importance. Addressing these uncertainties in an adequate way, providing insights into uncertainties, for example regarding underlying data and the registrations, were not addressed in the past Dutch BoDs, but will be a challenge for future Dutch BoD studies. Since its introduction the concept of DALY received criticism [40, 41] which is also applicable to the Dutch DALYs. A metric that has a hybrid manner of combining incidence approach for mortality and prevalence approach for morbidity, results in difficulties interpreting the outcomes. Promoting its concept, being transparent about data and methodology and good communication of the BoD results, for example through visualization, are activities that demand continuing efforts, also in the Dutch NBoD study.

The choice for the Dutch Life tables instead of a golden standard, results in outcomes which are less comparable with other countries, but most likely reflects the health loss in the Netherlands best. A similar argumentation for comparison can be raised regarding the disability weights. Using a Dutch study instead of generic disability weights gives most likely a good representation of the severity of morbidity in a Dutch cultural and health care context, but it hampers the comparability with the results of other NBoDs or the GBD. Also, having updated disability weights in the GBD, even partly based on recent research in the Netherlands [42], might call for an update. In addition, the underlying severity distributions of the various disease stages also require to be updated. Improvements in diagnoses and treatment might have changed these underlying distributions. However, available data to estimate these severity distributions are still limited.

For various health conditions, the Dutch YLD are based on registration data such as GP registrations which might be an underestimation of the population DALY. On the other hand, the correction for multimorbidity assumes independent occurrence of combinations of health conditions, while in reality they are not, given that many health conditions have shared risk factors. This might result in an overestimation of the YLD. Because of these uncertainties the DALY results are rather referred to as estimates of the burden of disease. Nevertheless, these estimates provide a picture of current status and future changes in the burden of disease. Being able to compare different health conditions, looking into the two components YLD and YLL and seeing how burden of disease might evolve over time provides valuable inputs for policy makers, for example to prioritize public health policy.

## Conclusion

The National Institute for Public Health and the Environment (RIVM) has over 20 years of experience in doing BoD estimates, and bringing them into the policy process. Results of BoD analyses are included in several national memoranda on health policy that the Dutch Ministry of Health, Welfare and Sport publishes every 4 years (e.g. [43, 44]) and projections of BoD have been used to formulate missions for the Dutch Cabinet [39].

Over the 20 years of experience, many improvements have been made along the way, such as the expansion of the selection of health conditions, the accounting for multimorbidity, methodology to estimate the total burden of disease and not only of a particular selection of health conditions, and the future projections of the burden of disease. These results found their way into the Dutch policy making processes.

Doing a NBoD study is not an effort undertaking easily. It requires a substantial amount of data, methodological choices, interpretation and presentation of results, and the personnel capacity to carry out all these tasks. The capacity building within our Public Health Institute has been an important precondition to safeguard continuity. Nevertheless, the process of understanding one’s national data, trying to improve the weaknesses, calculating DALYs, knowing the consequences of methodological choices, and interpreting and presenting results to e.g. policy makers, can have an enormous additional value in population health information and thus support better public health policies. It is not always the destination that counts, also its journey of getting there is sometimes equally important.

## Supplementary information

**Additional file 1: Supplementary Table 1.** Ten most important health conditions in 2015, ranked by DALYs, including other indicators.

**Additional file 2.**

## Data Availability

All data has been obtained from various data sources (see text in the article). Most of these data sources (such as vital statistics and cancer registration data) are publicly available. These Burden of Disease results are made available in an Excel document as supplementary material.

## Abbreviations

BoD: Burden of Disease
COPD: Chronic Obstructive Pulmonary Disease
DALY: Disability-Adjusted Life Years
EQ-5D: Standardized instrument for measuring generic health status.
GBD: Global Burden of Disease
GP: General Practitioners
ICD-10: 10th revision of the International Statistical Classification of Diseases and Related Health Problems
NBoD: National Burden of Disease
RIVM: Dutch National Institute for Public Health and the Environment
YLD: Years Lost due to Disability
YLL: Years of Life Lost

## References

[1] Murray CJ (1994). Quantifying the burden of disease: the technical basis for disability-adjusted life years. Bull World Health Organ.

[2] Ruwaard D, Kramers PGN. Public Health Status and Forecasts 1997 (PHSF 1997). The sum of the parts. [De som der delen]. RIVM-report 431501018. Utrecht: Elsevier/De Tijdstroom; 1997.

[3] Melse JM, Essink-Bot ML, Kramers PG, Hoeymans N (2000). A national burden of disease calculation: Dutch disability-adjusted life-years. Dutch burden of disease group. Am J Public Health.

[4] Oers JAM van. Public Health Status and Forecasts 2002 (PHSF 2002). Health on track? [Gezondheid op koers?] RIVM-report 270551001. Houten: Bohn Stafleu Van Loghum; 2002.

[5] de Hollander AEM, Hoeymans NN, Melse JM, van Oers JAM, Polder JJ (2007). Care for health. The 2006 Dutch public health status and forecasts report.

[6] van der Lucht F, Polder JJ (2010). From Health to Better [Van gezond naar beter] RIVM.

[7] Hoeymans N, Van Loon AJM, Van den Berg M, Harbers MM, Hilderink HBM, Van Oers JAM (2014). A healthier Netherlands: Key findings from the Dutch 2014 Public Health Status and Foresight Report.

[8] RIVM (2018). Public health foresight report 2018 - a healthy prospect.

[9] RIVM (2018). What diseases will we have in the future?.

[10] Devleesschauwer B, Praet N, Dorny P, Duchateau L, Speybroeck N. DALY calculation in practice: a stepwise approach. [Abstract] Eur J Public Health. 2013;23(suppl_1):ckt126.324. 10.1093/eurpub/ckt126.324. 10.1007/s00038-014-0553-y 24748107

[11] RIVM. Public health and health care Bilthoven: National Institute for Public Health and the Environment; 2020. Available from: https://www.volksgezondheidenzorg.info/onderwerp/english/introduction.

[12] Gijsen R, Poos M, Treurniet HF, GP W (1999). Selectie van ziekten en aandoeningen voor de Volksgezondheid Toekomkst Verkenningen. [Selection of diseases and conditions for the Public Health Status and Foresight studies] RIVM Rapport 278610001.

[13] Gijsen R, Poos M, Slobbe L, Mulder M, in ‘t Panhuis – Plasmans M, Hoeymans N (2013). Een nieuwe selectie van ziekten voor de Volksgezondheid Toekomst Verkenningen. RIVM Briefrapport 010003004/20s13.

[14] World Health Organization (2004). ICD-10 : international statistical classification of diseases and related health problems: tenth revision.

[15] RIVM. Methodology for Trend Scenario for Dutch Public Health Foresight Report Bilthoven: National Institute for Public Health and the Environment; 2018. Available from: https://www.vtv2018.nl/sites/default/files/2018-11/20181108%20Methodology%20Trend%20Scenario%20VTV2018%20Version%202.pdf.

[16] van Lier A, McDonald SA, Bouwknegt M, Kretzschmar ME, Havelaar AH, group EPI (2016). Disease Burden of 32 Infectious Diseases in the Netherlands, 2007–2011. PLoS One.

[17] Schroeder SA (2012). Incidence, prevalence, and hybrid approaches to calculating disability-adjusted life years. Popul Health Metrics.

[18] Havelaar AH, Haagsma JA, Mangen MJ, Kemmeren JM, Verhoef LP, Vijgen SM (2012). Disease burden of foodborne pathogens in the Netherlands, 2009. Int J Food Microbiol.

[19] Stouthard MEA, Essink-Bot ML, Bonsel GJ, Barendregt JJM, Kramers PGN, van de Water H (1997). Disability weights for diseases in the Netherlands.

[20] The EuroQol Group (1990). EuroQol - a new facility for the measurement of health-related quality of life. Health Policy.

[21] Devlin NJ, Brooks R (2017). EQ-5D and the EuroQol group: past, present and future. Appl Health Econ Health Policy.

[22] Melse JM, Kramers PGN (1998). Calculations of the burden of disease in the Netherlands. Background report for the Public Health Status and Forecast 1997.

[23] Mathers CD, Vos T, Lopez AD, Salomon JA, Ezzati M (2001). National Burden of Diseases Studies: A Practical Guide. Edition 2.0.

[24] Van Den Akker M, Buntinx F, Knottnerus JA (1996). Comorbidity or multimorbidity: What's in a name? A review of literature. Eur J Gen Pract.

[25] Hilderink HBM, Plasmans MH, Snijders BE, Boshuizen HC, Poos MJ, van Gool CH (2016). Accounting for multimorbidity can affect the estimation of the burden of disease: a comparison of approaches. Arch Public Health.

[26] IHME. Global Burden of Disease Study 2017 (GBD 2017) Reference Life Table 2019. Available from: http://ghdx.healthdata.org/record/global-burden-disease-study-2017-gbd-2017-reference-life-table.

[27] WHO (2016). Ill-defined causes in cause-of-death registration.

[28] Stolpe S. Regional differences in the use of ill-defined causes of death in cardiovascular mortality. [Abstract] Eur J Public Health. 2017;27(suppl_3):ckx187.711. 10.1093/eurpub/ckx187.711.

[29] IHME (2018). Determining causes of death: How we reclassify miscoded deaths.

[30] Statistics Netherlands (2019). Deaths among the Dutch population by main primary causes of death, age (at time of death) and sex.

[31] Harteloh P (2020). The implementation of an automated coding system for cause-of-death statistics. Inform Health Soc Care.

[32] IKNL (2020). Netherlands Cancer Registry.

[33] Nielen MMJ, Spronk I, Davids R, Korevaar JC, Poos R, Hoeymans N (2019). Estimating morbidity rates based on routine electronic health Records in Primary Care: observational study. JMIR Med Inform.

[34] Meerding WJ, Polinder S, Lyons RA, Petridou ET, Toet H, van Beeck F (2010). How adequate are emergency department home and leisure injury surveillance systems for cross-country comparisons in Europe?. Int J Inj Control Saf Promot.

[35] Landelijke Basisregistratie Ziekenhuiszorg (LBZ). Dutch Hospital Data 2020. Available from: https://www.dhd.nl/producten-diensten/lbz/Paginas/dataverzameling-lbz.aspx (Accessed 29 Apr 2020).

[36] Elnegaard S, Andersen RS, Pedersen AF, Larsen PV, Søndergaard J, Rasmussen S (2015). Self-reported symptoms and healthcare seeking in the general population -exploring “the symptom iceberg”. BMC Public Health.

[37] Plasmans MHD. Projecting causes of deaths in the Netherlands, an envelope approach. [Abstract] Eur J Public Health. 2017;27(suppl_3):ckx187.142, 10.1093/eurpub/ckx187.412.

[38] Statistics Netherlands (2016). Kernprognose 2016–2060 (Population Forecast 2016–2060).

[39] Health Holland (2019). Dutch Cabinet establishes 25 missions for the future: 5 address Health & Care.

[40] Anand S, Hanson K (1997). Disability-adjusted life years: a critical review. J Health Econ.

[41] Solberg CT, Norheim OF, Barra M (2018). The disvalue of death in the global burden of disease. J Med Ethics.

[42] Haagsma JA, Maertens de Noordhout C, Polinder S, Vos T, Havelaar AH, Cassini A (2015). Assessing disability weights based on the responses of 30,660 people from four European countries. Popul Health Metrics.

[43] MoH. National Health Policy Memorandum (Landelijke nota gezondheidsbeleid) 2020–2024. https://www.rijksoverheid.nl/binaries/rijksoverheid/documenten/rapporten/2020/02/29/gezondheid-breed-op-de-agenda/gezondheid-breed-op-de-agenda.pdf. (in Dutch): Ministry of Health, Welfare and Sport; 2020.

[44] MoH. Langer Gezond Leven. Ook een kwestie van gezond gedrag [Longer healthy living. Longer healthy life. Also a matter of healthy behavior]. https://zoek.officielebekendmakingen.nl/kst-22894-20-b1.pdf. (in Dutch): Ministry of Health Welfare and Sport; 2003.

