# Supplementary material for: Dutch DALYs, current and future burden of disease in the Netherlands
Source: Arch Public Health. 2020 Sep 22;78:85. doi: 10.1186/s13690-020-00461-8 (PMC7510132; doi:10.1186/s13690-020-00461-8)
Supplement: Supplementary file 1 — Additional file 1: Supplementary Table 1. Ten most important health conditions in 2015, ranked by DALYs, including other indicators. [file 13690_2020_461_MOESM1_ESM.docx]

Supplementary Table 1. Ten most important health conditions in 2015, ranked by DALYs, including other indicators

| Disease | DALY | YLD | YLL | Deaths | Occurrence |
| --- | --- | --- | --- | --- | --- |
| Coronary heart diseases | 260,200 | 164,000 | 96,200 | 9,000 | 732,200 |
| Stroke | 228,200 | 141,800 | 86,400 | 9,660 | 437,000 |
| Diabetes | 188,900 | 159,900 | 29,000 | 2,800 | 1,111,000 |
| COPD | 182,500 | 108,400 | 74,100 | 6,900 | 607,300 |
| Anxiety disorders | 173,900 | 173,900 | - | - | 1,046,300 |
| Lung cancer | 169,200 | 5,900 | 163,300 | 10,400 | 24,100 |
| Mood disorders | 160,400 | 158,600 | 1,900 | 100 | 551,600 |
| Neck and back complaints | 145,800 | 144,700 | 1,200 | 100 | 1,982,300 |
| Dementia | 129,800 | 45,700 | 84,000 | 13,800 | 154,000 |
| Osteoarthrosis | 129,100 | 127,900 | 1,200 | 200 | 1,199,100 |
